# Association between COVID-19 pandemic and school refusal among elementary school children in Japan: difference-in-differences analysis

**DOI:** 10.3389/fpubh.2025.1466209

**Published:** 2025-05-21

**Authors:** Tomoko Mizuno, Nobutoshi Nawa, Aya Isumi, Satomi Doi, Tomohiro Morio, Takeo Fujiwara

**Affiliations:** ^1^Department of Public Health, Institute of Science Tokyo, Tokyo, Japan; ^2^Department of Pediatrics and Developmental Biology, Institute of Science Tokyo, Tokyo, Japan; ^3^Department of Health Policy, Institute of Science Tokyo, Tokyo, Japan

**Keywords:** COVID-19, school refusal, difference in difference analysis, longitudinal study, mental health, Japan

## Abstract

**Background:**

The coronavirus disease 2019 (COVID-19) pandemic has been reported to have affected children's mental health. However, the impact of the COVID-19 pandemic on school refusal remains unclear. This study examined the association between the early stage of the COVID-19 pandemic and school refusal among elementary school children in Japan using the difference-in-differences approach.

**Methods:**

Data from the Adachi Child Health Impact of Living Difficulty study were used. We followed up with children without COVID-19 pandemic experience in the fourth grade in 2016 and the sixth grade in 2018 (control group, *n* = 449) and children with COVID-19 pandemic experience in the fourth grade in 2018 and the sixth grade in 2020 (COVID-19 group, *n* = 3,733).

**Results:**

Approximately 3.8 and 4.0% of students in the sixth grade in the control and COVID-19 groups, respectively, were school refusal. Change in the prevalence of school refusal from the fourth to the sixth grade in the control and COVID-19 groups was 2.4 and 2.0%, respectively. The difference-in-differences approach showed that the experience of the COVID-19 pandemic did not increase the risk of school refusal, which remained consistent even after performing propensity score matching.

**Conclusions:**

The early stage of the COVID-19 pandemic may not be associated with the risk of school refusal in elementary school children in Japan. A more long-term and comprehensive analysis is required to examine the full impact of the pandemic on school refusal.

## 1 Introduction

School refusal is defined as children's refusal or reluctance to go to school (1, 2), which is a precursor to school absenteeism (3). School refusal may be a manifestation of emotional problems, such as depression and anxiety (2), and a significant issue leading to school absenteeism (3). In Japan, the Ministry of Education, Culture, Sports, Science and Technology (MEXT) defines school absenteeism as a student's absence for 30 or more days due to psychological, emotional, physical, or social factors, excluding reasons such as illness, economic hardship, or avoidance of infection (4). School absenteeism has also become a significant issue in other countries (5). It can lead to emotional and social developmental impairment and poor academic performance in children (6) and is a risk factor for unemployment, marital problems, and mental illness in adults (7). Thus, school refusal is a vital indicator that allows caregivers or school teachers to intervene with children before school absenteeism occurs. Examining the risks of school refusal is critical.

The risk factors for school refusal are diverse (8). In their meta-analysis, Gubbles et al. (9) identified significant and substantial effects for the following risks related to school refusal: a child's physical and mental problems (e.g., psychiatric symptoms, depression, and anxiety), substance abuse, antisocial or risky behavior, problems at school, school characteristics, parenting problems and difficulties, and family problems. It has also has been reported that sleep problems, such as insomnia, parasomnia, and daytime sleepiness, are associated with school refusal (10).

According to MEXT data, in 2019, the leading causes of school absenteeism among elementary school children nationwide were as follows: 20.9% involved school-related factors (e.g., friendship issues, poor academic performance, relationships with teachers, and bullying), 22.9% were associated with family-related factors (e.g., parent–child relationships and sudden changes in the home environment), and 51.7% were due to factors involving the children themselves (e.g., anxiety, apathy, disruption of daily rhythms, and delinquency) (11). Additionally, a report from major school absenteeism treatment centers in Hiroshima Prefecture, Japan, indicated that the leading cause of school absenteeism in the elementary school group was parent–child relationship issues linked to separation anxiety (12). The characteristic causes of school absenteeism in Japan may include psychological pressure from parental expectations, an education system that prioritizes academic performance, and the challenges of adapting to group life.

Meanwhile, several reports indicate that the early stage of the coronavirus disease 2019 (COVID-19) pandemic has impacted children's mental health and sleep (13–15). For example, Xie et al. (16) found that 22.6% of children in second to sixth grades reported depressive symptoms in a survey conducted between February and March 2020 in China, which is higher than in previous surveys, and 18.9% reported anxiety symptoms. Gassman-Pines et al. (17) reported that daily survey data collected between February and April 2020 in the UK showed that the COVID-19 pandemic worsened the psychological wellbeing of both parents and children, especially in families facing multiple crises, such as job loss, income loss, caregiving burdens, and illness. Additionally, Lin et al. (18) conducted an online survey in China in February 2020 and reported that insomnia was more severe in women, young individuals, those living in the epicenter, and those experiencing a high threat from COVID-19. A Japanese study based on COVID-19-related data collected from July to August 2021 revealed that sleep-related problems, including insomnia, sleep debt, and delayed bedtime, may be associated with school refusal among adolescents (19). Furthermore, a systematic review indicated that several social restrictions aimed at preventing the spread of COVID-19, such as the closure of in-person schools, public areas, and playgrounds, increased internet addiction and online gaming disorders (20).

Considering the reported risk factors for school refusal, along with the COVID-19 pandemic worsening mental health and sleep issues, school refusal may potentially rise due to the pandemic. A report from the UK indicates that unauthorized absences and severe absenteeism in primary and secondary schools steadily increased prior to the 2019/2020 school year. In the 2022/2023 school year, the overall absenteeism rate was 7.5%, up from about 5% before the pandemic. Furthermore, the rate of persistent absenteeism (defined as missing over 10% of academic sessions) constituted 22.3% of total absenteeism, nearly double the pre-pandemic rate. The authors conclude that the pandemic has heightened emotionally based school avoidance (21).

The first case of COVID-19 was confirmed in Japan in January 2020. The nation experienced its first wave from February to June 2020, followed by a second wave from July to October 2020. The Japanese government mandated the closure of all elementary, junior high, and high schools during the first wave to prevent further spread of COVID-19 (22). For most schools, mandatory closures began on March 2, 2020, and continued until the end of May. According to MEXT, the number of school absentees among elementary students in Japan was 35,032 (0.54%) in 2017, 44,841 (0.70%) in 2018, 53,350 (0.83%) in 2019, and 63,350 (1.0%) in 2020 (4). Since school absenteeism had been increasing yearly even before the pandemic, the pandemic's impact on school refusal in children has not been fully elucidated. The trends in school refusal attributed to the pandemic in Japan should be assessed to establish a more substantial evidence-based support system. Thus, this study examined the association between the early stage of the COVID-19 pandemic and changes in school refusal among elementary school children in Japan. Additionally, possible risk factors for school refusal were assessed.

## 2 Materials and methods

### 2.1 Participants

**Figure 1:**
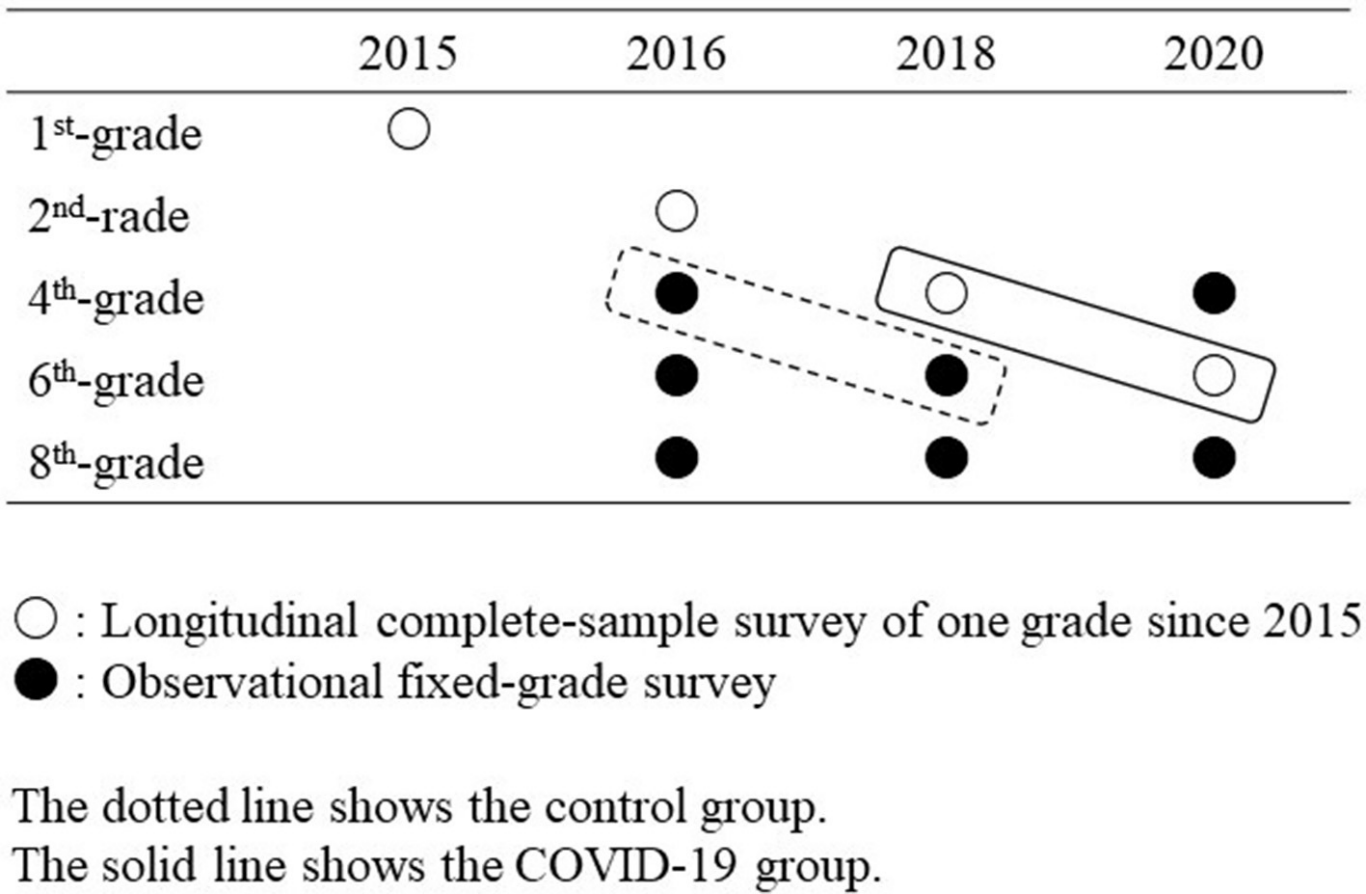
Target grade and survey schedule in Adachi Child Health Impact of Living Difficulty (ACHILD) study.

We used data from the Adachi Child Health Impact of Living Difficulty (A-CHILD) study, a population-based multiple cohort study conducted in public elementary schools in Adachi City, Tokyo, Japan (23). Adachi City is one of the 23 special wards in Tokyo Prefecture, with a population of ~690,000. The data were obtained from self-reported questionnaires distributed by teachers to students in schools. If a student was absent, the school offered an opportunity to distribute the questionnaire over several weeks. The children took the questionnaire home for their caregivers to complete. The children submitted the completed questionnaires anonymously to the school in envelopes. The A-CHILD study comprises two cohorts. The baseline survey, a complete-sample survey, was conducted on first graders (aged 6–7 years) in all 69 elementary schools in the city in 2015, with follow-up surveys performed in 2016 and every 2 years thereafter. Additionally, a survey targeting fourth graders in nine elementary schools across five administrative areas in the city has been conducted every 2 years since 2016. Therefore, two waves of panel data from fourth graders (aged 9–10 years) to sixth graders (aged 11–12 years) were available in the following cohorts: children in the fourth grade in 2018 and the sixth grade in 2020, and children in the fourth grade in 2016 and the sixth grade in 2018 (Figure 1).

**Figure 2:**
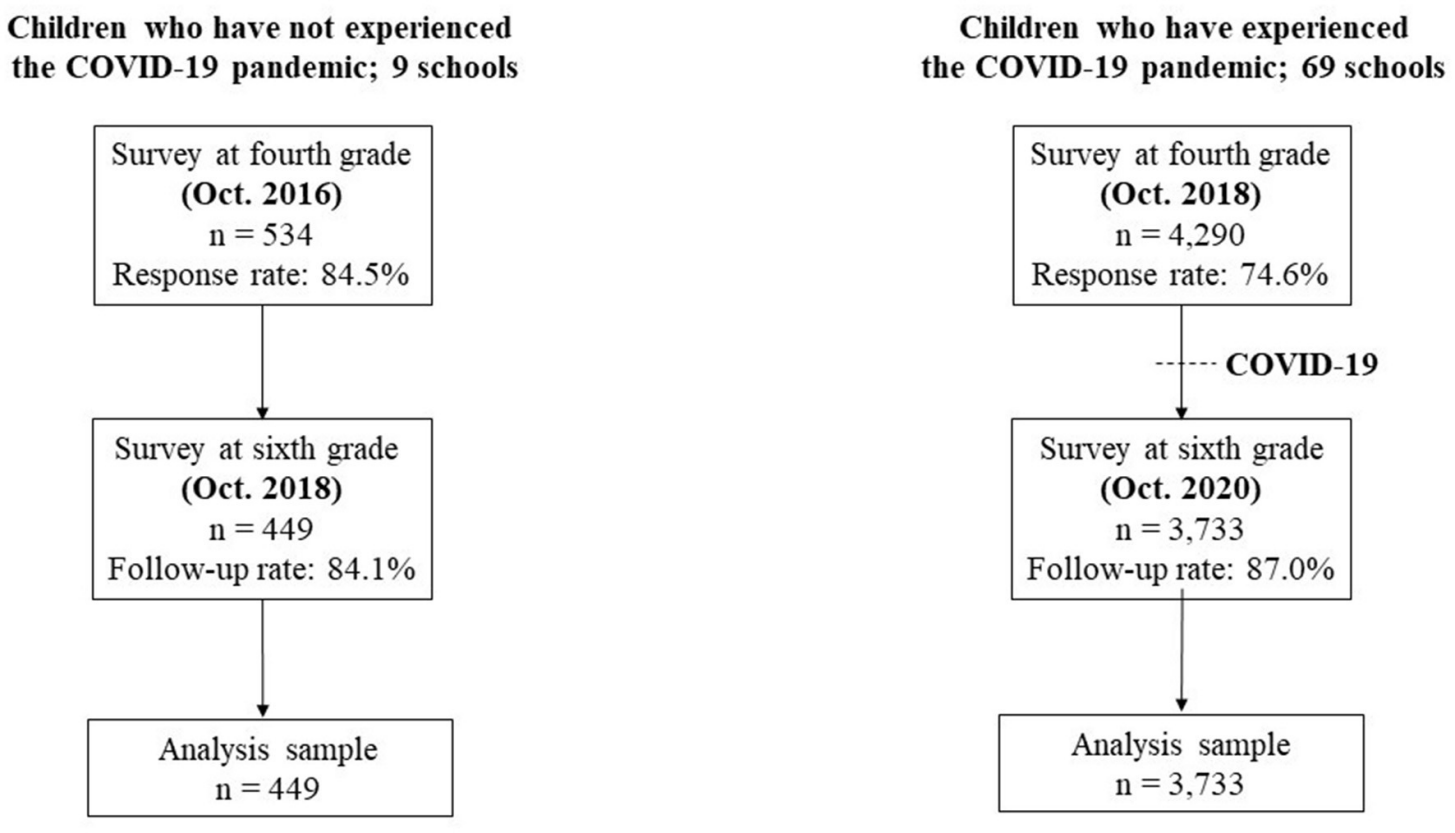
Flowchart of the study participants.

Figure 2 presents the flowchart for participant inclusion in the current study. In 2016, informed consent was gathered from 534 children and caregivers in the fourth grade across nine elementary schools (response rate: 86.7%), and 449 were followed up in the sixth grade in 2018 (follow-up rate: 84.1%). This group did not experience the pandemic (control group). Additionally, we conducted a survey in 2018 involving fourth graders and their caregivers from 69 public elementary schools. Informed consent was obtained from 4,290 children and caregivers (response rate: 80.8%), and 3,733 were followed up in the sixth grade in 2020 (follow-up rate: 87.0%). This group experienced the pandemic in 2020 (COVID-19 group). Japanese elementary schools start in April, with each survey conducted in October. Furthermore, during the survey conducted in October 2020, remote learning was not being implemented in Adachi City.

### 2.2 Measurements

#### 2.2.1 School refusal

Caregivers were asked whether their child had been absent from school at the beginning of the fourth and sixth grades. They were also questioned about the reasons for the child's school absence based on the following categories: (1) illness or injury, (2) family reasons, (3) the child did not want to go to school, and (4) other. School refusal was defined as responding to category (3) and being absent for >1 day. The response was dichotomized (0 = no, 1 = yes). We coded participants who had missing answers to the school absenteeism questions as not having school refusal.

#### 2.2.2 Demographic characteristics of the participants

Caregivers were asked about the child's sex (boy or girl), birth order (eldest or not), the caregiver's marital status (married or single/divorced/bereaved/others), annual household income (< 3.0 or ≥3.0 million JPY; 110 JPY = 1 USD), the parent's educational status (high school graduate or more or not high school graduate), and the mental health of the caregiver. The caregiver's mental health was assessed using the Japanese version of the Kessler 6 (K6) (24). The K6 comprises six items related to depression and anxiety, which are scored from 0 to 24. Moderate psychological distress is defined as a score of 5–12, while severe psychological distress is defined as a score of ≥13 (25). The responses were dichotomized (< 5 or ≥5).

#### 2.2.3 Lifestyle factors

Caregivers were asked about the child's lifestyle factors, including bedtime, wake-up time, breakfast, tooth brushing frequency, and time spent using mobile phones. For the logistic regression analysis, the responses in each question were dichotomized or combined into the following categories: wake-up time, before or after 7 am; bedtime, before or after 10 pm; have breakfast daily or not everyday; frequency of tooth brushing, more than twice a day or less than once a day; and time spent on using mobile phone, < 1 h, 1 to < 2 h, or ≥2 h.

#### 2.2.4 Child mental health

Child emotional and behavioral problems- including emotional symptoms, conduct issues, hyperactivity/inattention, and peer problems- were assessed using the Japanese version of the Strengths and Difficulties Questionnaire (SDQ) developed by Robert Goodman (26). The total difficulty score ranges from 0 to 40. A score of < 13 is categorized as typical, 13 to < 16 as borderline, and ≥16 as clinical. Previous studies have reported the reliability and validity of the SDQ in Japanese children (27, 28).

Child resilience was assessed using the Children's Resilient Coping Scale (CRCS) (29). The scale included eight items with high internal consistency (Cronbach's alpha > 0.80). Caregivers rated the child's resilience and coping behavior on a scale from 0 (never) to 4 (very frequently). The total CRCS score was derived from the sum of the eight items (range: 0–32). Higher total scores indicated greater resilience. A score below the 10th percentile was classified as low resilience and dichotomized (0 = not low or 1 = low).

### 2.3 Statistical analysis

Using the chi-square test, we first evaluated whether the change in the mean of each time-varying variable from fourth to sixth grade significantly differed between the COVID-19 and control groups. Specifically, the mean total scores for the SDQ, CRCS, and K6 were used to determine if the changes in each score from fourth to sixth grade significantly varied between the two groups. Only child bedtime exhibited a significant difference between the two groups among all the variables. Consequently, the analysis used the bedtime, caregiver marital status, and parental K6 as covariates. The reason for including caregiver marital status and parental K6 is that previous studies have reported their association with school refusal (7, 30).

We then applied the multiple imputation approach under the assumption of missing at random to minimize potential bias due to missing information. We generated 50 imputed datasets using the multiple imputation with chained equations procedure. The covariates for imputation included bedtime, marital status, and parental K6. For the prediction of these variables, we used those without missing values: school ID, sex, birth order, school refusal status, and group. We compared the data distribution between the two groups before and after imputation using the generated imputed datasets and confirmed no significant fluctuations in the estimates. The analysis results from all the imputed datasets were combined using Rubin's rules for multiple imputations (31).

We next examined the association between variables measured in the fourth grade and school refusal in the sixth grade in the control and COVID-19 groups through logistic regression analysis. In addition to the results of the crude model analysis, the results of Model 1 were also presented, where all variables were included in the model simultaneously. Among all variables, only annual household income was excluded from the adjusted variables due to a variance inflation factor, indicating multicollinearity >10.

The difference-in-differences approach was then performed to compare changes in the prevalence of school refusal from the fourth to sixth grade between the control and COVID-19 groups using the significance level on the interaction term of the group based on grades. The model included individual fixed effects. The models took the following form:


$$\begin{array}{l} y_{ij}=\beta_{0}+\beta_{1}Cohort_{i}+\beta_{2}Grade_{ij}+\beta_{3}Cohort_{i}^{*}Grade_{ij}+AW_{i}\\ \;+BX_{ij}+\;e_{ij}, \end{array}$$

where *y*_*ij*_ denotes school refusal for a child *i* in grade *j, cohort* is an indicator variable that takes the value of zero for the control group and one for the COVID-19 group, *grade* is an indicator variable that takes the value of zero for the observation in the fourth grade and one for the observation in the sixth grade, *W* denotes individual fixed effects, and *X* indicates time-varying covariates. Under the parallel trend assumption, β_3_ was interpreted as the causal effect of the COVID-19 pandemic on school refusal. The following models were constructed to examine whether the measured time-varying factors explained the association: a crude model, a model adjusting for covariates such as marital status and parental K6 (Model 1), and a model adjusting for bedtime, which was a possible mediating variable in addition to the covariates in Model 1 (Model 2).

Finally, propensity score (PS) matching was performed to compare the characteristics between the control and the COVID-19 groups. Multiple logistic regression models calculated individual PSs incorporating the following covariates from the fourth and sixth grades: bedtime, marital status, and parental K6. We conducted a 1:1 optimal propensity score matching within a 0.001 caliper width without replacement. The balance of covariates between the matched pairs was assessed based on standardized biases, which were less than 10% for all variables and not significant in a chi-squared test (see Supplementary Table 1). Using the matched pairs, the difference-in-differences analysis was conducted again. STATA version 15.0 (Stata Corp LLC, College Station, Texas) was used for all analyses.

## 3 Results

**Figure 3:**
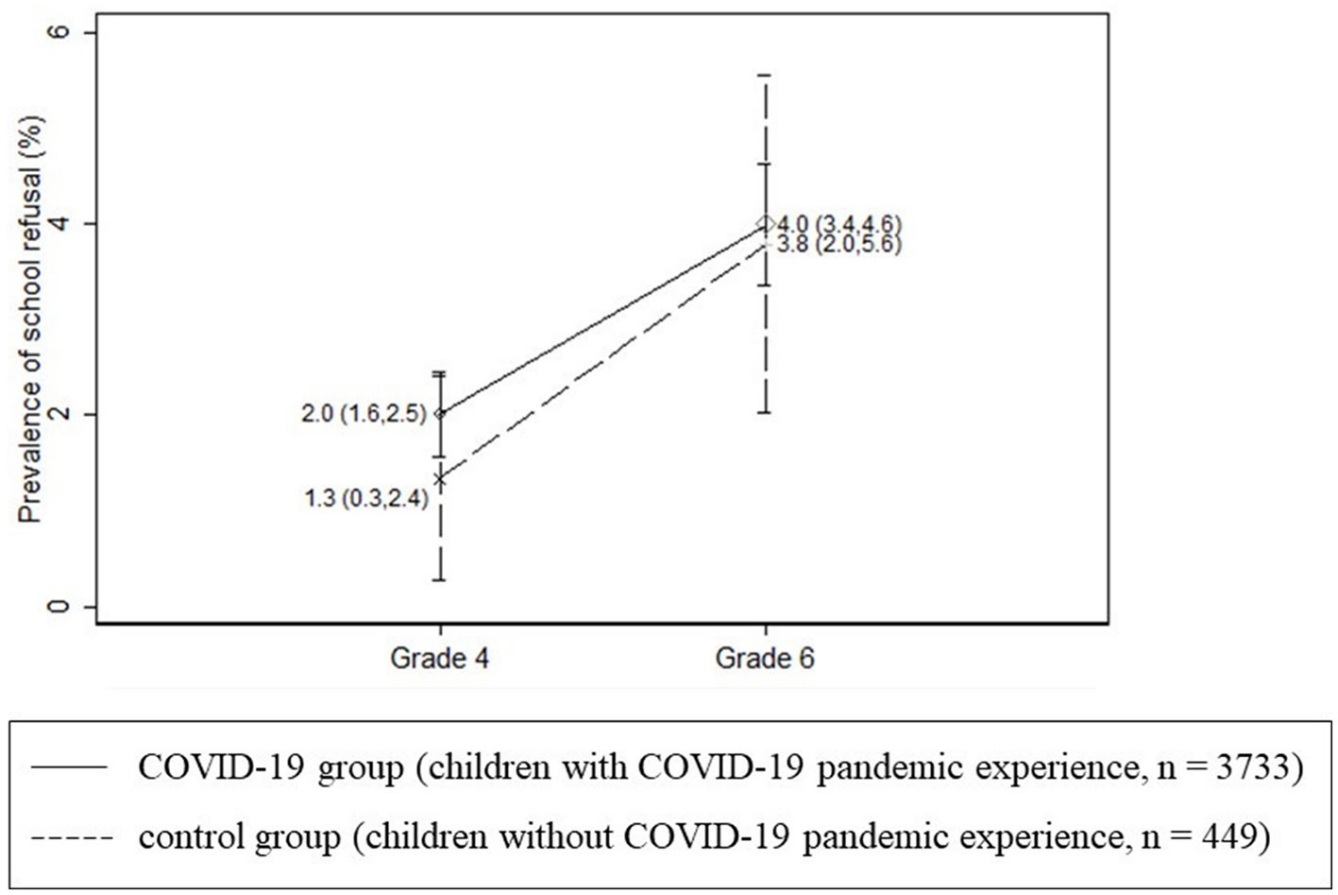
Average and 95% confidence interval of the prevalence of school refusal by grade and group.

In the control group (*n* = 449), 1.3% (*n* = 6) and 3.8% (*n* = 17) of children in the fourth and sixth grades, respectively, refused to attend school. In the COVID-19 group (*n* = 3,733), 2.0% (*n* = 75) and 4.0% (*n* = 149) of children in the fourth and sixth grades, respectively, refused to attend school (Figure 3).

Table 1 shows the demographic characteristics of the control and COVID-19 groups in the fourth and sixth grades. In the fourth grade, the COVID-19 group used mobile phones for longer periods per day (hours) than did those in the control group (16.8% vs. 11.4%).

**Table 1:** Demographic characteristics of the study participants without and with COVID-19 pandemic experience (control group and COVID-19 group).

**School year**	**4th grade**			**6th grade**		
**Group**	**Children without COVID-19 pandemic experience (*****N** =* **449)**	**Children with COVID-19 pandemic experience (*****N** =* **3,733)**	***p*** **-value**	**Children without COVID-19 pandemic experience (*****N** =* **449)**	**Children with COVID-19 pandemic experience (*****N** =* **3,733)**	***p*** **-value**
	***N*** **(%)**	***N*** **(%)**		***N*** **(%)**	***N*** **(%)**	
**Time invariant variables**						
**Child's sex**						
Male	235 (52.3%)	1,869 (50.1%)	0.36			
Female	214 (47.7%)	1,864 (49.9%)				
**Father's educational status**						
High school graduate or more	379 (84.4%)	2,441 (65.4%)	0.99			
Not high school graduate	37 (8.2%)	239 (6.4%)				
Missing	33 (7.4%)	1,053 (28.2%)				
**Mother's educational status**						
High school graduate or more	405 (90.2%)	2,650 (71.0%)	0.97			
Not high school graduate	25 (5.6%)	162 (4.3%)				
Missing	19 (4.2%)	921 (24.7%)				
**Time varying variables**						
**Annual household income (JPY)**						
< 3 million	48 (10.7%)	366 (9.8%)	0.89	47 (10.5%)	359 (9.6%)	0.66
≥3 million	366 (81.5%)	2,852 (76.4%)		355 (79.0%)	2,914 (78.1%)	
Unknown/missing	35 (7.8%)	515 (13.8%)		47 (10.5%)	460 (12.3%)	
**Marital status**						
Married	389 (86.6%)	2,828 (75.8%)	0.34	385 (85.8%)	2,799 (75.0%)	0.34
Single/divorced/bereaved/others	44 (9.8%)	272 (7.3%)		50 (11.1%)	312 (8.3%)	
Missing	16 (3.6%)	633 (16.9%)		14 (3.1%)	622 (16.7%)	
**Birth order**						
Eldest	154 (34.3%)	1,210 (32.4%)	0.42	148 (32.9%)	1,211 (32.4%)	0.82
Not eldest	295 (65.7%)	2,523 (67.6%)		301 (67.1%)	2,522 (67.6%)	
**Wake-up time**						
< 7 am	253 (56.3%)	2,045 (54.8%)	0.56	271 (60.4%)	1,956 (52.4%)	**0.001**
≥7 am	195 (43.4%)	1,671 (44.7%)		174 (38.7%)	1,765 (47.3%)	
Missing	1 (0.2%)	17 (0.5%)		4 (0.9%)	12 (0.3%)	
**Bedtime**						
< 10 pm	260 (57.9%)	2,087 (55.9%)	0.33	149 (33.2%)	953 (25.5%)	**< 0.001**
≥10 pm	182 (40.5%)	1,615 (43.3%)		294 (65.5%)	2,752 (73.7%)	
Missing	7 (1.6%)	31 (0.8%)		6 (1.3%)	28 (0.8%)	
**Having breakfast**						
Everyday	407 (90.6%)	3.379 (90.5%)	0.83	394 (87.7%)	3,263 (87.4%)	0.77
Not everyday	40 (8.9%)	320 (8.6%)		51 (11.4%)	442 (11.8%)	
Missing	2 (0.5%)	34 (0.9%)		4 (0.9%)	28 (0.8%)	
**Toothbrushing**						
≥ twice a day	352 (78.4%)	2,873 (77.0%)	0.59	371 (82.6%)	3,171 (84.9%)	0.59
≤ once a day	88 (19.6%)	769 (20.6%)		70 (15.6%)	555 (14.9%)	
Missing	9 (2.0%)	91 (2.4%)		8 (1.8%)	7 (0.2%)	
**Using mobile phone (per day)**						
< 1 h	300 (66.8%)	2,145 (57.4%)	**< 0.001**	181 (40.3%)	1,187 (31.8%)	**< 0.001**
1–2 h	90 (20.0%)	936 (25.1%)		124 (27.6%)	943 (25.3%)	
≥2 h	51 (11.4%)	627 (16.8%)		139 (31.0%)	1,566 (41.9%)	
Missing	8 (1.8%)	25 (0.7%)		5 (1.1%)	37 (1.0%)	
**SDQ**						
TDS < 13	330 (73.5%)	2,698 (72.3%)	0.79	344 (76.6%)	2,920 (78.2%)	0.82
TDS ≥ 13, < 16	55 (12.3%)	405 (10.9%)		44 (9.8%)	340 (9.1%)	
TDS ≥ 16	62 (13.8%)	507 (13.6%)		49 (10.9%)	393 (10.5%)	
Missing	2 (0.5%)	123 (3.3%)		12 (2.7%)	80 (2.2%)	
TDS (mean, SD)	9.65 (5.18)	9.33 (5.54)		8.75 (5.30)	8.54 (5.29)	
**CRCS**						
Not low	400 (89.1%)	3,348 (89.4%)	0.77	399 (88.9%)	3,290 (88.1%)	0.72
Low	48 (10.7%)	383 (10.3%)		50 (11.1%)	436 (11.7%)	
Missing	1 (0.2%)	2 (0.3%)		0 (0.0%)	7 (0.2%)	
Total score (mean, SD)	22.02 (4.92)	22.24 (5.25)		22.93 (4.98)	22.89 (5.25)	
**Parental psychological distress**						
K6 < 5	285 (63.5%)	2,454 (65.7%)	0.08	284 (63.2%)	2,439 (65.3%)	0.30
K6 ≥ 5	162 (36.1%)	1,161 (31.1%)		158 (35.2%)	1,216 (32.6%)	
Missing	2 (0.4%)	118 (3.2%)		7 (1.6%)	78 (2.1%)	
Total score (mean, SD)	4.03 (4.31)	3.77 (4.30)		4.11 (4.23)	3.95 (4.46)	

Bold indicates p < 0.05.

TDS, total difficulty score.

Additionally, a notable difference in bedtimes was observed from the fourth to sixth grade between the COVID-19 and control groups (*p* = 0.022). The COVID-19 group had a higher percentage of participants with bedtimes after 10 pm compared to the control group (from 43.3% to 73.7% vs. 40.5% to 65.5%).

Table 2 presents the association between the variables measured in the fourth grade and refusal to attend school in the sixth grade in the control and COVID-19 groups using logistic regression analysis after multiple imputations. In Model 1 in the control group, being unmarried [odds ratio (OR): 6.06, 95% confidence interval (CI): 1.25–29.4] and a high parental K6 score at the fourth grade (OR: 3.06, 95% CI: 0.98–9.58) were associated with a greater risk of school refusal at the sixth grade (*p* < 0.05). In the COVID-19 group, having a father who did not graduate from high school (OR: 1.90, 95% CI: 1.07–3.39), not having breakfast daily (OR: 1.74, 95% CI: 1.08–2.82), using a mobile phone for >2 h (OR: 1.52, 95% CI: 1.04–2.33) a, high SDQ score (OR: 1.92, 95% CI: 1.21–3.05), low CRCS score (OR: 1.62, 95% CI: 1.03–2.56), a high parental K6 score (OR: 1.60, 95% CI: 1.10–2.31), and school refusal in the fourth grade (OR: 8.95, 95% CI: 5.08–15.8) were all associated with a higher risk of school refusal in the sixth grade (*p* < 0.05).

**Table 2:** Association between variables measured in the fourth grade and school refusal in the sixth grade by logistic regression analysis among children without COVID-19 pandemic experience (control group, *N* = 449) and children with COVID-19 pandemic experience (COVID-19 group, *N* = 3,733) after multiple imputation.

**Group**	**Children without COVID-19 pandemic experience (***N =* **449)**		**Children with COVID-19 pandemic experience (***N =* **3,733)**	
**Variables in 4th grade**	**Crude OR (95% CI)**	**Model 1 OR (95% CI)**	**Crude OR (95% CI)**	**Model 1 OR (95% CI)**
**Child sex**				
Male	Ref	Ref	Ref	Ref
Female	1.25 (0.47, 3.29)	1.28 (0.42, 3.93)	0.88 (0.64, 1.23)	0.98 (0.70, 1.39)
**Father's educational status**				
High school graduate	Ref	Ref	Ref	Ref
Not high school graduate	1.75 (0.38, 8.12)	1.79 (0.28, 11.3)	**2.34 (1.38, 3.97)**	**1.90 (1.07, 3.39)**
**Mother's educational status**				
High school graduate	Ref	Ref	Ref	Ref
Not high school graduate	2.62 (0.56, 12.3)	1.01 (0.12, 8.40)	1.71 (0.88, 3.35)	1.01 (0.48 2.15)
**Annual household income (JPY)**				
≥3 million	Ref		Ref	
< 3 million	**8.76 (2.92, 26.2)**		1.55 (0.96, 2.50)	
**Marital status**				
Married	Ref	Ref	Ref	Ref
Single/divorced/bereaved/others	**3.86 (1.29, 11.5)**	**6.06 (1.25, 29.4)**	1.34 (0.75, 2.39)	0.70 (0.31, 1.59)
**Birth order**				
Eldest	Ref	Ref	Ref	Ref
Not eldest	0.96 (0.35, 2.63)	0.46 (0.14, 1.51)	1.28 (0.88, 1.84)	1.09 (0.74, 1.62)
**Wake-up time**				
< 7 am	Ref	Ref	Ref	Ref
≥ 7 am	1.31 (0.48, 3.56)	0.74 (0.22, 2.47)	1.25 (0.90, 1.74)	0.92 (0.64, 1.33)
**Bedtime**				
< 10 pm	Ref	Ref	Ref	Ref
≥ 10 pm	1.88 (0.69, 5.15)	1.47 (0.43, 4.95)	**1.58 (1.13, 2.21)**	1.23 (0.84, 1.80)
**Having breakfast**				
Everyday	Ref	Ref	Ref	Ref
Not everyday	**6.35 (2.21, 18.2)**	3.02(0.72, 12.6)	**2.88 (1.90, 4.38)**	**1.74 (1.08, 2.82)**
**Toothbrushing**				
≥ twice a day	Ref	Ref	Ref	Ref
≤ once a day	2.50 (0.88, 7.08)	2.37 (0.68, 8.30)	1.27 (0.86, 1.87)	0.94 (0.62, 1.43)
**Using mobile phone (per day)**				
< 1 h	Ref	Ref	Ref	Ref
1–2 h	1.90 (0.62, 5.83)	1.37 (0.36, 5.18)	1.13 (0.75, 1.72)	1.09 (0.71, 1.67)
≥ 2 h	2.02 (0.53, 7.73)	0.86 (0.13, 5.77)	**2.27 (1.54, 3.35)**	**1.52 (1.04, 2.33)**
**SDQ**				
TDS < 13	Ref	Ref	Ref	Ref
TDS ≥ 13, < 16	1.85 (0.49, 6.93)	1.56 (0.34, 7.21)	**2.07 (1.27, 3.37)**	1.63 (0.97, 2.73)
TDS ≥ 16	1.63 (0.43, 6.09)	0.47 (0.08, 2.59)	**3.33 (2.26, 4.92)**	**1.92 (1.21, 3.05)**
**CRCS**				
Not low	Ref	Ref	Ref	Ref
Low	**4.11 (1.36, 12.4)**	3.33 (0.81, 13.6)	**2.85 (1.92, 4.23)**	**1.62 (1.03, 2.56)**
**Parental psychological distress**				
K6 < 5	Ref	Ref	Ref	Ref
K6 ≥ 5	**2.79 (1.01, 7.70)**	**3.06 (0.98, 9.58)**	**2.17 (1.54, 3.06)**	**1.60 (1.10, 2.31)**
**School refusal in 4th grade**				
No	NA	NA	Ref	Ref
Yes	NA	NA	**12.4 (7.36, 20.9)**	**8.95 (5.08, 15.8)**

Model 1: all variables were put into the model simultaneously.

Annual household income was omitted from the adjusted covariates due to multicollinearity of VIF > 10.

Bold indicates p < 0.05.

OR, odds ratio; CI, confidence interval; TDS, total difficulty score.

Table 3 shows the results of the odds ratios for school refusal in the sixth grade among all children and children with and without COVID-19 pandemic experience. Additionally, the interaction between exposure to the COVID-19 pandemic and the grade analyzed using the difference-in-differences analysis is also shown. This is the interaction term when comparing the odds ratios of school refusal associated with participants going from the fourth to the sixth grade with and without COVID-19 pandemic experience. The children had a greater risk of school refusal in the sixth grade than in the fourth grade in the crude model (OR = 2.47, 95% CI: 1.82–3.35) and adjusted models (OR = 2.41, 95% CI: 1.75–3.32 in Model 1; OR = 2.25, 95% CI: 1.59–3.20 in Model 2). Since the OR did not change significantly after the adjustment for bedtime (i.e., 2.41 vs. 2.25), it is possible that bedtime is not a mediating factor The children without COVID-19 pandemic experience had a higher risk of school refusal in the sixth grade in the crude model (OR = 2.83, 95% CI: 1.12–7.19), but not in the adjusted models (OR = 2.46, 95% CI: 0.90–6.74 in Model 1; OR = 1.95, 95% CI: 0.66–5.78 in Model 2). In contrast, the children with COVID-19 pandemic experience had a greater risk of school refusal in the sixth grade in the crude model (OR = 2.42, 95% CI: 1.75–3.35) and adjusted models (OR = 2.38, 95% CI: 1.70–3.33 in Model 1; OR = 2.29, 95% CI: 1.57–3.34 in Model 2). The interaction term between being exposed to the COVID-19 pandemic and grade was not significant in any model (OR = 0.86, 95% CI: 0.32–2.29, OR = 0.97, 95% CI: 0.35–2.66, and OR = 0.95, 95% CI: 0.35–2.64, respectively), implying that the experience of the COVID-19 pandemic did not increase the odds ratios of school refusal associated with participants going from the fourth to the sixth grade (i.e., the experience of the COVID-19 pandemic is not an effect modifier).

**Table 3:** Odds ratios for school refusal in the sixth grade among all children, children with and without COVID-19 pandemic experience, respectively, and the interaction term between being exposed to COVID-19 and grade.

**Group**	**School year**	**Crude OR (95% CI)**	**Model 1 OR (95% CI)**	**Model 2 OR (95% CI)**
All children	4th	Ref	Ref	Ref
	6th	**2.47 (1.82, 3.35)**	**2.41 (1.75, 3.32)**	**2.25 (1.59, 3.20)**
Children without the COVID-19 pandemic experience (control group)	4th	Ref	Ref	Ref
	6th	**2.83 (1.12, 7.19)**	2.46 (0.90, 6.74)	1.95 (0.66, 5.78)
Children with the COVID-19 pandemic experience (COVID-19 group)	4th	Ref	Ref	Ref
	6th	**2.42 (1.75, 3.35)**	**2.38 (1.70, 3.33)**	**2.29 (1.57, 3.34)**
The interaction term between being exposed to COVID-19 pandemic and grade		0.86 (0.32, 2.29; *p* = 0.756)	0.97 (0.35, 2.66; *p* = 0.947)	0.95 (0.35, 2.64; *p* = 0.927)

Model 1: adjusting for marital status (time varying) and parental K6 (time varying).

Model 2: additionally adjusting for bedtime (time varying) in addition to covariates for Model 1.

Bold indicates p < 0.05.

OR, odds ratio; CI, confidence interval.

The analysis following PS matching is summarized in the Supplementary Tables. Supplementary Tables 1, 2 present the demographic characteristics in both fourth and sixth grades, with and without COVID-19 experience, before and after PS matching, respectively. After PS matching, 440 children were matched, and the three covariates were comparable between the two groups. Supplementary Table 3 presents the difference-in-differences analysis. The interaction term between exposure to the COVID-19 pandemic and grade was not significant in any model (OR = 1.04, 95% CI: 0.26–4.22, OR = 1.10, 95% CI: 0.26–4.68, and OR = 1.10, 95% CI: 0.26–4.81, respectively), and the outcome remained unchanged before PS matching.

## 4 Discussion

Our study examined whether the early stage of the COVID-19 pandemic led to school refusal among elementary school children using data from a multiple population-based longitudinal cohort study using the difference-in-differences method. We found that the early stage of the pandemic was not associated with an increased risk of school refusal in elementary school children in Japan. This result remained consistent even after performing PS matching. Instead, both the COVID-19 and control groups exhibited a similar rise in school refusal in the sixth grade compared to the fourth grade. To our knowledge, this is the first longitudinal study that explored the association between the COVID-19 pandemic and school refusal among children. Assessing the risks of school refusal accurately is crucial, as it may lead to school absenteeism.

Several studies have reported that the COVID-19 pandemic can cause anxiety, distress, and potentially depression among children due to fear of the disease, staying at home, limited outdoor activities and social interaction, change in sleep rhythms and lifestyles, and increased parental stress (13–19). These adverse effects may increase the risk of school refusal (9). In contrast, a study on primary and secondary school students in China found that participants were generally satisfied with their family life during the school closure period during the COVID-19 pandemic, and increased opportunities for parent—child discussions about COVID-19 may have alleviated their psychopathological symptoms (32). These positive effects may have contributed to the lack of a significant increase in school refusal during the early stage of the pandemic. Additionally, in Japan, including Adachi City, elementary schools were closed from March to May 2020 due to the pandemic. During this period, online remote learning had not started in Adachi City, and children were required to complete and submit assignments given by the school at home. Therefore, the children might have welcomed the reopening of schools. This survey was conducted shortly after the schools reopened in October 2020; thus, it is possible that school refusal had not yet become evident. The pandemic's impact on school refusal must be analyzed from a longer-term perspective. Furthermore, strengthening support systems through school counselors and increasing local mental health support services may help attenuate the rise in school refusal. Since no specific data were available to evaluate these factors in the current analysis, they were not included as covariates but should be considered for future studies.

We also found that both the COVID-19 and control groups experienced a similar increase in the rate of school refusal in the sixth grade compared with the fourth grade. This is consistent with a previous study that found school refusal increases in the higher grades (4). We also found that single-parent and parental psychological distress at the fourth grade were associated with school refusal at the sixth grade in the control group, while paternal education, not having breakfast daily, using mobile phones for more than 2 h, child behavior problems, resilience and parental psychological distress, and having refused to attend school in the fourth grade were associated with school refusal in the sixth grade. Previous studies have reported that poor mental health conditions in children, parental education, and parental depression are associated with the refusal to attend school (5, 30), which aligns with our findings. Moreover, a systematic review of the effect of breakfast showed that eating breakfast positively affects cognitive performance when compared to skipping it. It also showed the beneficial effects of school breakfast programs on increased attendance and reduced absenteeism (33). Although long-time usage of mobile phones has been reported to be a risk factor for behavioral or cognitive problems (34), no studies have reported an association between significant usage of mobile phones and refusal to attend school. In the COVID-19 group, the usage time of mobile phones in the fourth grade was longer than in the control group. Thus, the difference in usage time of mobile phones in the fourth grade may have led to the different findings for the risk of school refusal from mobile phone usage in the two groups. Additionally, in the COVID-19 group, child behavior problems and resilience among fourth graders were associated with school refusal in the sixth grade, which were not observed in the control group. It has been reported that the COVID- 19 pandemic may have exacerbated mental health issues in children who were already mentally vulnerable (35, 36); thus, children in the COVID-19 group who were mentally vulnerable may have been more affected and experienced a higher risk of school refusal. However, we found no link between COVID-19 exposure and school refusal, potentially because no significant changes in the risk factors were observed that could contribute enough to lead to school refusal due to the COVID-19 pandemic.

Our study had several limitations. First, school refusal was defined as being absent for more than one day because of not wanting to go to school, which might be too short a time to assess school refusal properly. While this is useful, as it can lead to school absenteeism, further long-term studies are needed to explore the association between COVID-19 and school absenteeism. Second, we did not determine the detailed reasons for not wanting to go to school. Kearney et al. (3) emphasized the importance of evaluating school refusal from multiple perspectives, including information from parents, teachers, and the students themselves. Hence, further evaluations should be performed to determine the association between the reasons for school refusal and the COVID-19 pandemic, which may facilitate a better understanding of the mechanisms of school refusal. Third, we assessed school refusal based on the caregiver's report. Thus, the outcome measurement might be misclassified, potentially underestimating the association. Moreover, we did not assess school absenteeism based on the official school records at the school. Hence, further study on attendance should be conducted using the official school record. Fourth, there is a large difference in the sample size between the COVID-19 and control groups, potentially affecting the result. Fifth, the difference-in-differences analysis is based on the parallel trend and common shock assumptions. That is, in the absence of the intervention, the trends in outcomes for the treatment and control groups would have followed a similar path over time. There is also no difference between the treatment and control groups in the occurrence or nonoccurrence of nonintervention events. If such events do occur for both groups, there is no difference in their effects between the treatment and control groups (37). In our data, confirming the parallel trend assumption is not feasible because the observational data were measured at only two points in time, not at multiple time points. To address this issue, we performed PS matching and confirmed consistent results. However, the COVID-19 and control groups were not exactly similar; there may have been external factors affecting only one of the groups, or there may have been potential confounding factors. If so, our analysis might have been biased in assessing the impact of the COVID-19 pandemic on school refusal.

Despite these limitations, our longitudinal study found no association between the early stage of the COVID-19 pandemic and school refusal among elementary school children according to population-based data. The public health implication is that the recent rise in school refusal reported by some news media (38, 39) may not be linked to the COVID-19 pandemic, at least during the early stage examined in this study. Using a difference-in-differences analysis, we demonstrated that the increase in school refusal might simply be attributed to the progression of the school year, rather than the pandemic. This study analyzed data from the early stage of the pandemic and had methodological limitations. Therefore, a more long-term and comprehensive analysis is required to examine the full impact of the pandemic on school refusal.

## 5 Conclusion

In conclusion, the early stage of the COVID-19 pandemic may not be associated with the risk of school refusal in elementary school children in Japan. A long-term study should be conducted to better understand the link between the COVID-19 pandemic and children's refusal to attend school.

## Data Availability

The original contributions presented in the study are included in the article/Supplementary Material, further inquiries can be directed to the corresponding author.
