# Supplementary material for: Association between COVID-19 pandemic and school refusal among elementary school children in Japan: difference-in-differences analysis
Source: Front Public Health. 2025 May 21;13:1466209. doi: 10.3389/fpubh.2025.1466209 (PMC12133766; doi:10.3389/fpubh.2025.1466209)
Supplement: Supplementary file 1 [file Table_1.docx]

**Supplementary TABLE 1**

Demographic characteristics in the fourth grade without and with COVID-19 experience before and after propensity score matching

|  | Before PS matching | | | | After PS matching | | | |
| --- | --- | --- | --- | --- | --- | --- | --- | --- |
| Group | Children without COVID-19  pandemic experience  (N=449) | Children with COVID-19 pandemic experience  (N=3,733) | *p*-value | Bias  (%) | Children without  COVID-19  pandemic experience  (N=440) | Children with  COVID-19 pandemic experience  (N=440) | *p*-value | Bias  (%) |
|  | N (%) | N (%) |  |  | N (%) | N (%) |  |  |
| Variables |  |  |  |  |  |  |  |  |
| Marital status |  |  | 0.343 |  |  |  | 0.855 |  |
| Married | 389 (86.6%) | 2828 (75.8%) |  |  | 383 (87.1%) | 378 (85.9%) |  |  |
| Single/divorced/bereaved/others | 44 (9.8%) | 272 (7.3%) |  | -10.0 | 42 (9.5%) | 47 (10.7%) |  | 4.1 |
| Missing | 16 (3.6%) | 633 (16.9%) |  | 43.5 | 15 (3.4%) | 15 (3.4%) |  | 0.0 |
|  |  |  |  |  |  |  |  |  |
| Bedtime |  |  | 0.326 |  |  |  | 0.998 |  |
| < 10 pm | 260 (57.9%) | 2087 (55.9%) |  |  | 257 (58.4%) | 258 (58.6%) |  |  |
| ≥ 10 pm | 182 (40.5%) | 1615 (43.3%) |  | 3.2 | 179 (40.7%) | 178 (40.5%) |  | -0.5 |
| Missing | 7 (1.6%) | 31 (0.8%) |  | -6.1 | 4 (0.9%) | 4 (0.9%) |  | 0.0 |
|  |  |  |  |  |  |  |  |  |
| Parental psychological distress |  |  | 0.080 |  |  |  | 0.914 |  |
| K6 < 5 | 285 (63.5%) | 2454 (65.7%) |  |  | 283 (64.3%) | 289 (65.7%) |  |  |
| K6 ≥ 5 | 162 (36.1%) | 1161 (31.1%) |  | -9.4 | 156 (35.5%) | 150 (34.1%) |  | -2.9 |
| Missing | 2 (0.4%) | 118 (3.2%) |  | 9.8 | 1 (0.2%) | 1 (0.2%) |  | 0.0 |
| Total score (mean, SD) | 4.03 (4.31) | 3.77 (4.30) |  |  | 4.01 (4.33) | 3.99 (4.40) |  |  |

Bold indicates *p* < 0.05

**Supplementary TABLE 2**

Demographic characteristics in the sixth grade without and with COVID-19 experience before and after propensity score matching

|  | Before PS matching | | | | After PS matching | | | |
| --- | --- | --- | --- | --- | --- | --- | --- | --- |
| Group | Children without COVID-19  pandemic experience  (N=449) | Children with COVID-19 pandemic experience  (N=3,733) | *p*-value | Bias  (%) | Children without  COVID-19  pandemic experience  (N=440) | Children with  COVID-19 pandemic experience  (N=440) | *p*-value | Bias  (%) |
|  | N (%) | N (%) |  |  | N (%) | N (%) |  |  |
| Variables |  |  |  |  |  |  |  |  |
| Marital status |  |  | 0.344 |  |  |  | 0.946 |  |
| Married | 385 (85.8%) | 2799 (75.0%) |  |  | 382 (86.8%) | 379 (86.1%) |  |  |
| Single/divorced/bereaved/others | 50 (11.1%) | 312 (8.3%) |  | -10.7 | 44 (10.0%) | 47 (10.7%) |  | 2.3 |
| Missing | 14 (3.1%) | 622 (16.7%) |  | 45.0 | 14 (3.2%) | 14 (3.2%) |  | 0.0 |
|  |  |  |  |  |  |  |  |  |
| Bedtime |  |  | **<0.001** |  |  |  | 0.882 |  |
| < 10 pm | 149 (33.2%) | 953 (25.5%) |  |  | 144 (32.7%) | 151 (34.3%) |  |  |
| ≥ 10 pm | 294 (65.5%) | 2752 (73.7%) |  | 16.9 | 292 (66.4%) | 285 (64.8%) |  | -3.5 |
| Missing | 6 (1.3%) | 28 (0.8%) |  | -7.7 | 4 (0.9%) | 4 (0.9%) |  | 0.0 |
|  |  |  |  |  |  |  |  |  |
| Parental psychological distress |  |  | 0.30 |  |  |  | 0.997 |  |
| K6 < 5 | 284 (63.2%) | 2439 (65.3%) |  |  | 279 (63.4%) | 278 (63.2%) |  |  |
| K6 ≥ 5 | 158 (35.2%) | 1216 (32.6%) |  | -4.4 | 155 (35.2%) | 156 (35.5%) |  | 0.5 |
| Missing | 7 (1.6%) | 78 (2.1%) |  | -12.3 | 6 (1.4%) | 6 (1.4%) |  | 0.0 |
| Total score (mean, SD) | 4.11 (4.23) | 3.95 (4.46) |  |  | 4.13 (4.25) | 4.17 (4.51) |  |  |

Bold indicates *p* < 0.05

**Supplementary TABLE 3**

Odds ratios for school refusal in the sixth grade among all children, children with and without COVID-19 pandemic experience, respectively, and the interaction term between being exposed to COVID-19 and grade, after propensity score matching.

|  |  | Crude  OR (95% CI) | Model 1  OR (95% CI) | Model 2  OR (95% CI) |
| --- | --- | --- | --- | --- |
| All children | 4th | Ref | Ref | Ref |
|  | 6th | **2.50 (1.28, 4.88)** | **2.54 (1.27, 5.09)** | **2.26 (1.09, 4.71)** |
| Children without the COVID-19 pandemic experience (control group) | 4th | Ref | Ref | Ref |
|  | 6th | 2.50 (0.97, 6.44) | 2.31 (0.84, 6.37) | 1.86 (0.63, 5.54) |
| Children with the COVID-19 pandemic experience (COVID-19 group) | 4th | Ref | Ref | Ref |
|  | 6th | 2.60 (0.93, 7.29) | 2.61 (0.98, 6.92) | 2.63 (0.88, 7.91) |
| The interaction term between being exposed to COVID-19 pandemic and grade |  | 1.00 (0.26, 3.81; *p*=1.000) | 1.21 (0.30, 4.93; *p*=0.788) | 1.12 (0.27, 4.64; *p*=0.871) |

Model 1: adjusting for using marital status (time varying) and parental K6 (time varying).

Model 2: additionally adjusting for bedtime (time varying) in addition to covariates for Model 1

Bold indicates *p* < 0.05.

OR, odds ratio; CI, confidence interval.
